# Supplementary material for: Identification of the KIF18A alpha-4 helix as a therapeutic target for chromosomally unstable tumor cells
Source: Front Mol Biosci. 2024 Feb 12;11:1328077. doi: 10.3389/fmolb.2024.1328077 (PMC10896213; doi:10.3389/fmolb.2024.1328077)
Supplement: Supplementary file 1 [file Image1.pdf]

Figure S1.

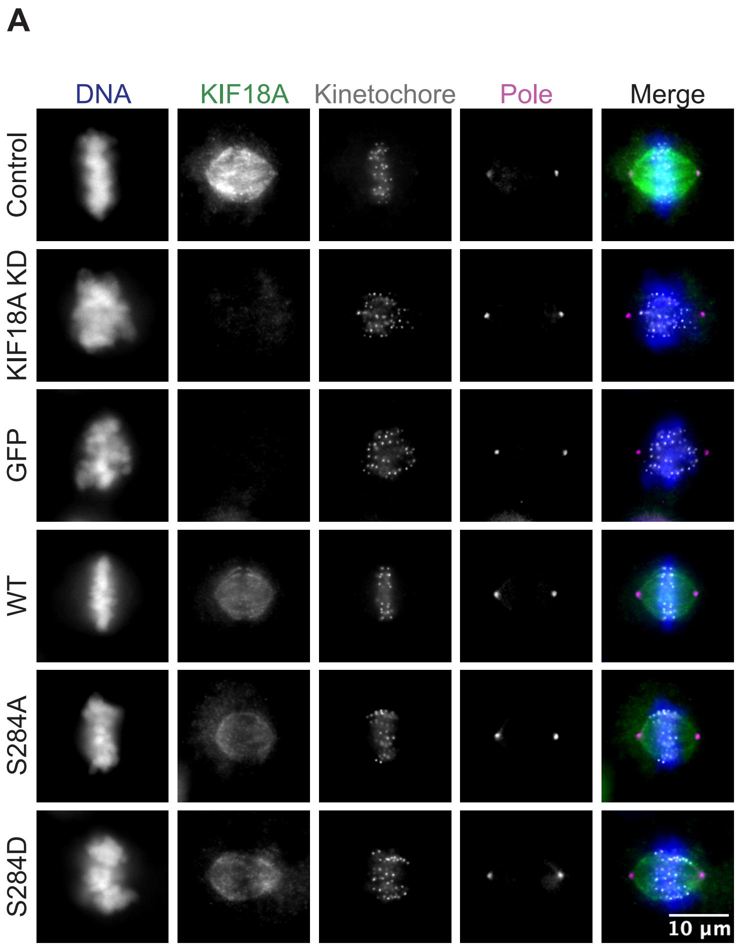
A
DNA
KIF18A
Kinetochore
Pole
Merge
Control
KIF18A KD
GFP
WT
S284A
S284D
10 µm

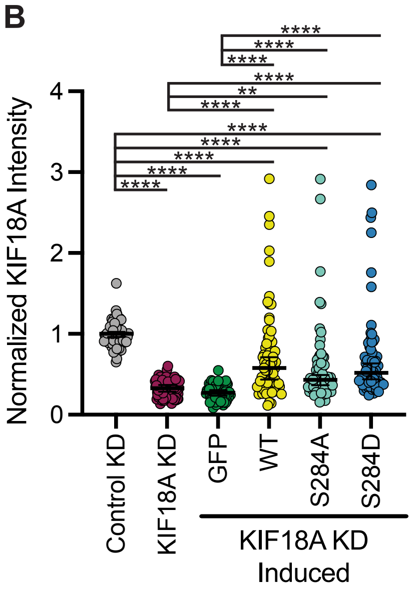
B
Normalized KIF18A Intensity
0
1
2
3
4
Control KD
KIF18A KD
GFP
WT
S284A
S284D
KIF18A KD
Induced
****
**

Figure S1: GFP-KIF18A S284 mutants are expressed at similar levels to wild type GFP-KIF18A. (A) Immunofluorescence images of HeLa Kyoto inducible cells fixed and stained 24 hours after endogenous KIF18A knockdown and induction of indicated GFP-KIF18A construct. Scale bar = 10 μm. Text colors indicate pseudo color in merged image. From left to right DAPI/DNA, KIF18A antibody staining, CENP-C antibody staining, γ-Tubulin antibody staining, merged image. **(B)** Quantification of KIF18A expression from KIF18A antibody staining. Fluorescence values were normalized to the mean control KIF18A intensity. Solid horizontal line indicates mean, vertical lines indicate standard deviation. Each dot represents a single cell. The total number of cells analyzed for each condition were control = 62, KIF18A KD = 80, GFP = 75, GFP-KIF18A WT = 68, GFP-S284A = 68, and GFP-S284D = 75. Data acquired from three experimental replicates. A one-way ANOVA with Tukey's test for multiple comparisons was run, P values: < 0.05 (*), < 0.01 (**), < 0.001 (***), <0.0001 (****). If no significance is indicated, result was not significant (> 0.05).

Figure S2.

A

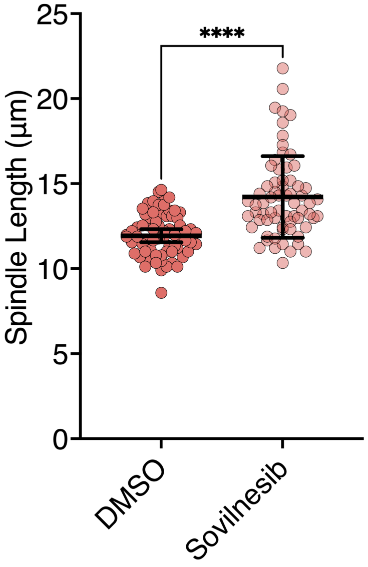

****
Spindle Length (µm)
25
20
15
10
5
0
DMSO
Sovilnesib

B

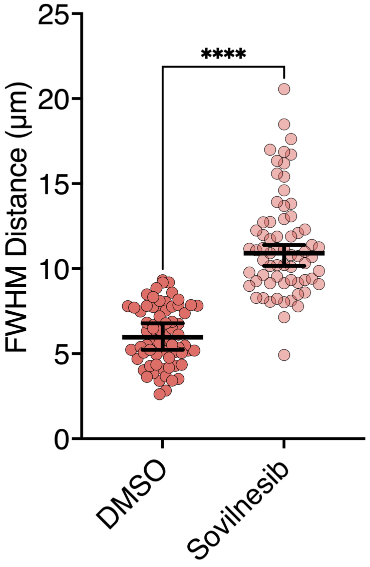

****
FWHM Distance (µm)
25
20
15
10
5
0
DMSO
Sovilnesib

Figure S2: Sovilnesib mimic KIF18A knockdown phenotypes in MDA-MB-231 cells. Graphs of spindle lengths (A) and full-width at half maximum (FWHM) of centromere fluorescence distribution along the length of the spindle (B) measured in cells fixed and stained 24 hours after the indicated treatments. Each dot represents a single cell (N= 68 for DMSO, N=67 for Sovilnesib). Mean ± standard deviation is displayed. Statistical results are shown for a Kruskal-Wallis with Dunn's Multiple Comparisons test. P value style: < 0.05 (*), < 0.01 (**), < 0.001 (***), <0.0001 (****). If no significance is indicated, result was not significant (> 0.05).

Figure S3.

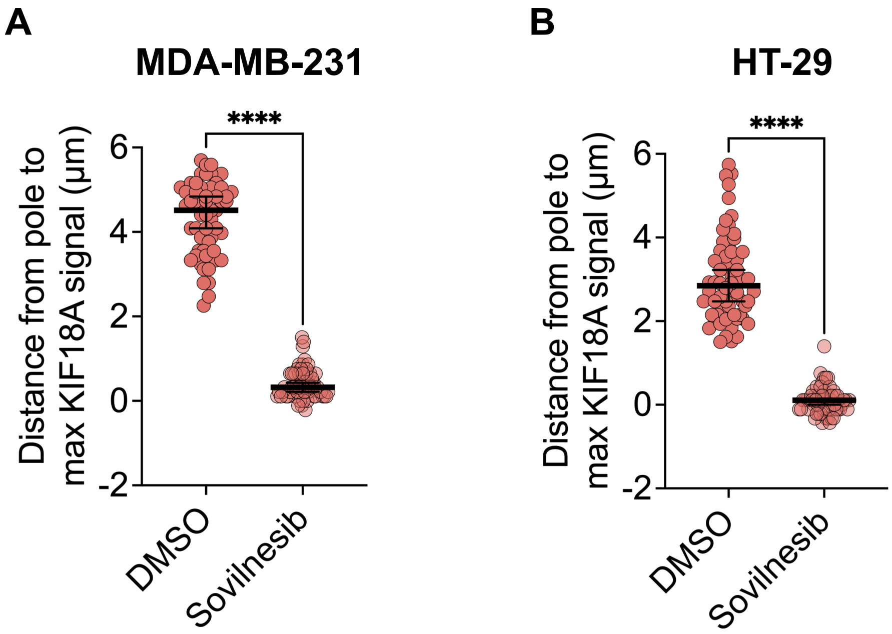
A
MDA-MB-231
****
Distance from pole to max KIF18A signal (μm)
6
4
2
0
-2
DMSO
Sovilnesib
B
HT-29
****
Distance from pole to max KIF18A signal (μm)
6
4
2
0
-2
DMSO
Sovilnesib

Figure S3: Sovilnesib disrupts microtubule plus-end localization of KIF18A. (A-B) Plots of distance from max KIF18A signal to the spindle pole derived from line scan analyses of KIF18A distribution in MDA-MB-231 **(A)** and HT-29 **(B)** cells. Each dot represents a single cell (MDA-MB-231: N=57 DMSO, N=60 Sovilnesib; HT-29: N=60 DMSO, N=60 Sovilnesib). Mean and SD are indicated by bars. Statistical results are shown for a Kruskal-Wallis with Dunn's Multiple Comparisons test. P value: < 0.05 (*), < 0.01 (**), < 0.001 (***), <0.0001 (****). If no significance is indicated, result was not significant (> 0.05).

Figure S4.

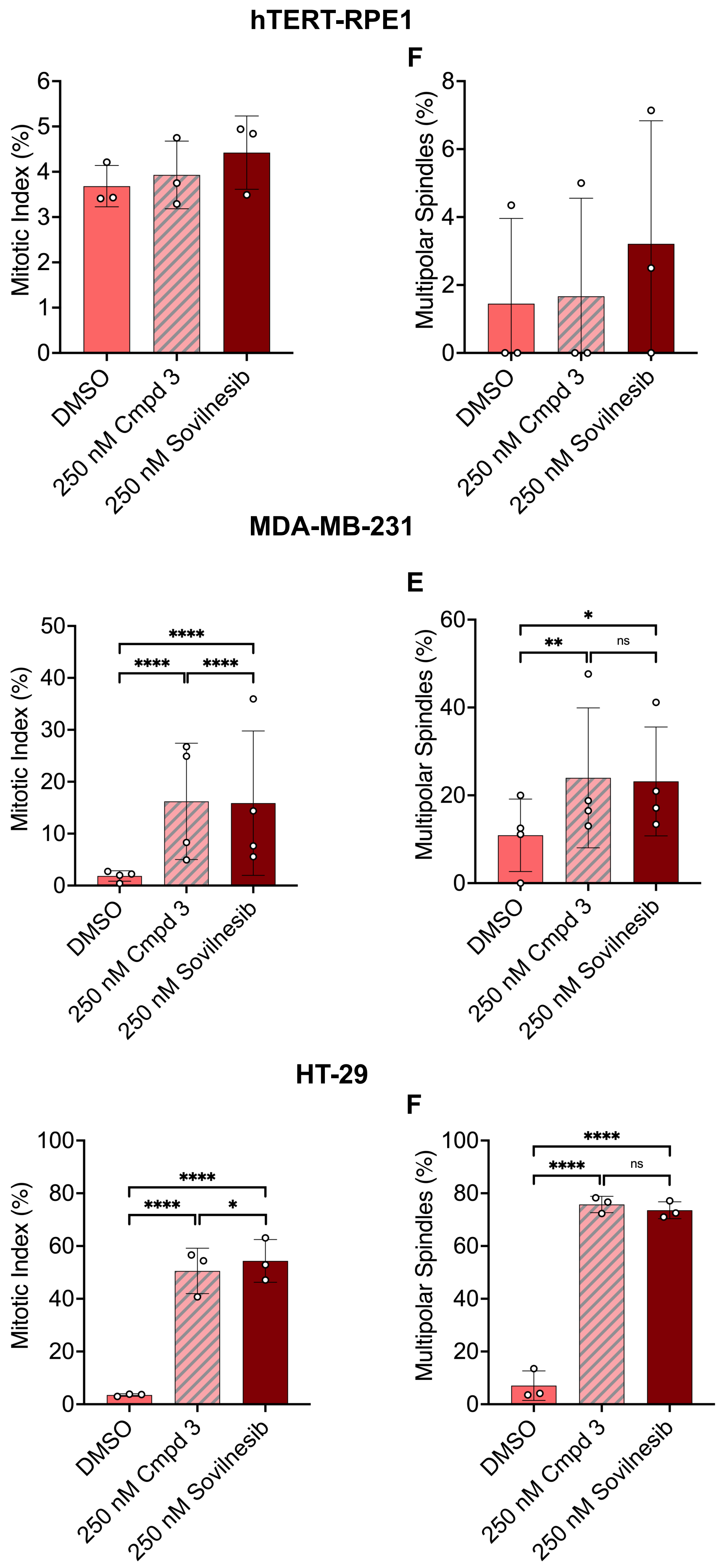

hTERT-RPE1
A
Mitotic Index (%)
0
1
2
3
4
5
6
DMSO
250 nM Cmpd 3
250 nM Sovilnesib
F
Multipolar Spindles (%)
0
2
4
6
8
DMSO
250 nM Cmpd 3
250 nM Sovilnesib
MDA-MB-231
B
Mitotic Index (%)
0
10
20
30
40
50
****
****
****
DMSO
250 nM Cmpd 3
250 nM Sovilnesib
E
Multipolar Spindles (%)
0
20
40
60
*
**
ns
DMSO
250 nM Cmpd 3
250 nM Sovilnesib
HT-29
C
Mitotic Index (%)
0
20
40
60
80
100
****
****
*
DMSO
250 nM Cmpd 3
250 nM Sovilnesib
F
Multipolar Spindles (%)
0
20
40
60
80
100
****
****
ns
DMSO
250 nM Cmpd 3
250 nM Sovilnesib

Figure S4: Sovilnesib treatment leads to mitotic arrest and multipolar spindles in chromosomally unstable breast and colorectal cancer cells. (A-C) Quantification of mitotic index (% of total cells in mitosis) in hTERT-RPE1 **(A)**, MDA-MB-231 **(B)**, and HT-29 cells **(C)** 24-hours after indicated treatments. Bars are mean ± standard deviation. Each dot indicates an experimental replicate. **(D-F)** Quantification of multipolar spindles (% of total spindles) in hTERT-RPE1 **(D)**, MDA-MB-231, **(E)** and HT-29 cells **(F)** 24 hours after indicated treatments. Bars are mean ± standard deviation. Each dot indicates an experimental replicate. Data were compared using a chi-square test. P value style: < 0.05 (*), < 0.01 (**), < 0.001 (***), <0.0001 (****). N.s. indicates not significant (> 0.05).

Figure S5.

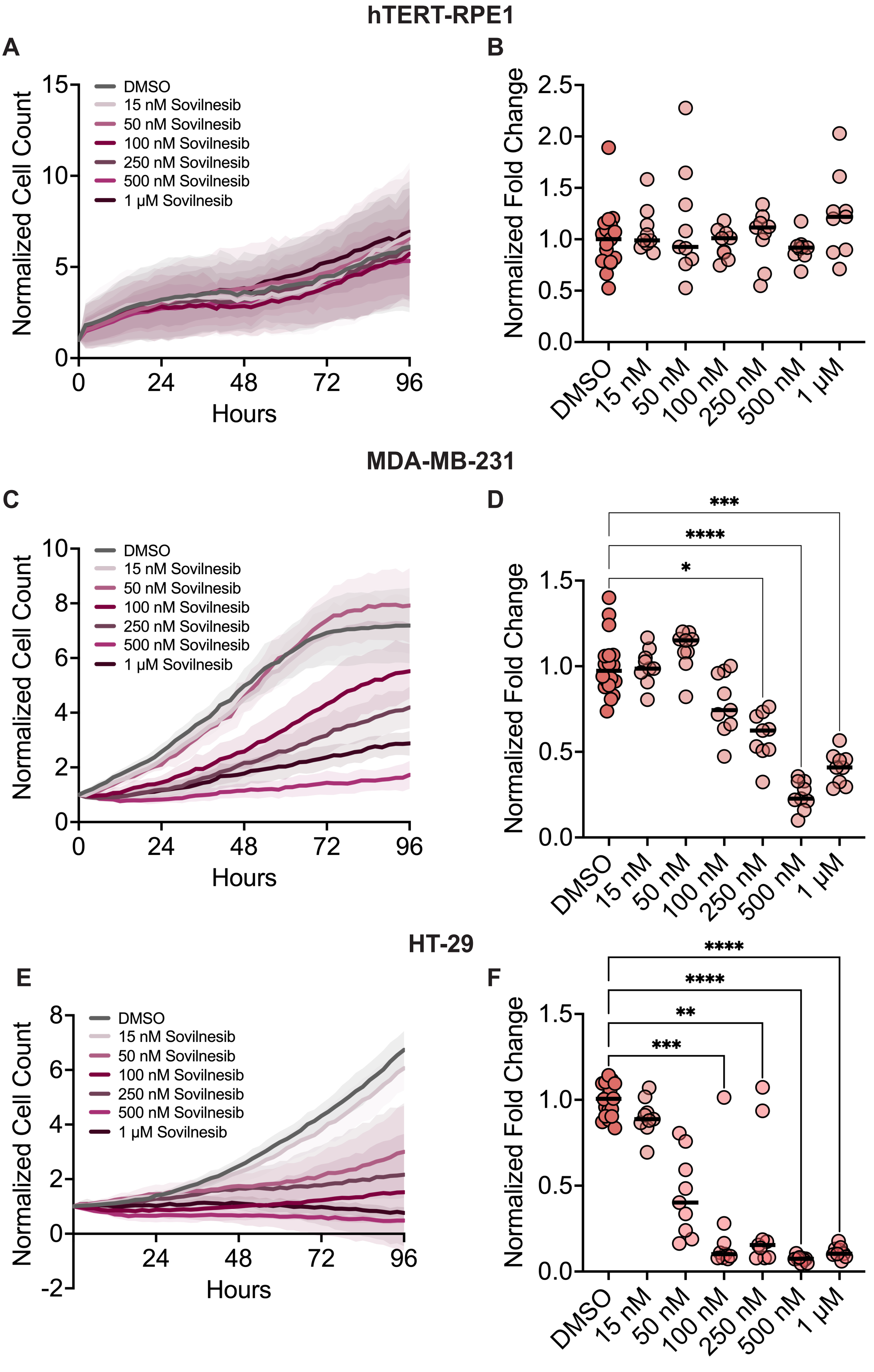

hTERT-RPE1
A
B
MDA-MB-231
C
D
HT-29
E
F
DMSO
15 nM Sovilnesib
50 nM Sovilnesib
100 nM Sovilnesib
250 nM Sovilnesib
500 nM Sovilnesib
1 µM Sovilnesib
Normalized Cell Count
Hours
Normalized Fold Change
DMSO
15 nM
50 nM
100 nM
250 nM
500 nM
1 µM
*
**
***
****

Figure S5: Sovilnesib reduces the proliferation of chromosomally unstable cells in a dose-dependent manner. (A) Plot of normalized cell count over time for cells treated with the indicated concentrations of Sovilnesib. Lines indicate mean and shaded area denotes SD. **(B)** Plot of normalized hTERT-RPE1 cell count (displayed as a % of DMSO control) as a function of Sovilnesib concentration. **(C)** Plot of normalized cell count over time for MDA-MB-231 cells treated with the indicated concentrations of Sovilnesib. Lines indicate mean and shaded area denotes SD. **(D)** Plot of normalized MDA-MB-231 cell count (displayed as a % of DMSO control) as a function of Sovilnesib concentration. **(E)** Plot of normalized cell count over time for HT-29 cells treated with the indicated concentrations of Sovilnesib. Lines indicate mean and shaded area denotes SD. **(F)** Plot of normalized HT-29 cell count (displayed as a % of DMSO control) as a function of Sovilnesib concentration. For plots in **(B)**, **(D)**, and **(F)**, each dot indicates an individual well and bars indicate mean values. Statistical results displayed from a Mann-Whitney test. P value style: < 0.05 (*), < 0.01 (**), < 0.001 (***), <0.0001 (****). If no significance is indicated, result was not significant (> 0.05). Data shown on all plots are from three independent experiments.
